# ASO Author Reflections: Effect Sizes of Whole Breast Radiotherapy and Systemic Therapies on Regional Recurrence Incidence in Breast Cancer Patients

**DOI:** 10.1245/s10434-020-08434-5

**Published:** 2020-04-04

**Authors:** Julia E. C. van Steenhoven, Thijs van Dalen

**Affiliations:** 1 Department of Surgery, Diakonessenhuis Utrecht, Utrecht, The Netherlands; 2 Department of Pathology, University Medical Center Utrecht, Utrecht, The Netherlands

## Past

Twenty years’ experience in practicing sentinel lymph node biopsy (SLNB) in breast cancer patients demonstrates that residual axillary tumor burden is not synonymous with disease recurrence over time. While meta-analyses have reported a false-negative rate (FNR) for SLNB between 5 and 7%, the reported regional recurrence (RR) rate in node-negative patients is much lower (0.3–0.6%).^1^ Moreover, landmark randomized controlled trials (RCTs) in patients who do have a tumor-positive SLNB but do not undergo completion axillary lymph node dissection (ALND) show that these patients will also rarely develop axillary recurrences despite an almost 30% chance of having residual positive lymph nodes.^2^ Apart from an National Surgical Adjuvant Breast and Bowel Project (NSABP) protocol B-04-like self-limiting phenomenon that we recently demonstrated in a study in SLNB N0 patients who had undergone ablative surgery without receiving further additional treatment and had a RR risk of only 2%,^3^ adjuvant radiotherapy (RT) and systemic treatments are factors that temper the growth of metastases in the axilla. In the present study, we aimed to quantify the effects of whole-breast RT and systemic treatments on the RR incidence in a large-population-based cohort of SLN N0 breast cancer patients.^4^

## Present

In our study comprising 13,512 patients staged as N0 according to SLNB, the cumulative 5-year RR was 1.4%.^4^ We demonstrated that RT, as a routine part of breast-conserving therapy (BCT), chemotherapy, and hormonal therapy independently exerted a mitigating effect on the risk of developing RR with hazard ratios of 0.46, 0.31, and 0.40, respectively. None of these treatments had been given with the intent to reduce risk of developing RR. This is the first study to report the magnitude of these effects in a large population-based cohort and may help to explain the observed discrepancy between the FNR of SLNB and RR in N0 patients. Extrapolating the effect size to the SLNB N+ patient category receiving whole-breast RT as routine part of BCT and commonly receiving adjuvant systemic treatment also helps to explain the gap between the rate of additional non-SLNs (27%) and the observed RR of 1.5% without axillary surgery.

## Future

The present data may help to explain the discrepancy between residual tumor-positive nodes and the observed RR risk. These data may also be of help to omit axillary surgery in patient categories for whom evidence is scarce. A trial including SLNB-positive patients who had undergone ablative surgery and were randomized to undergo or not axillary clearance was stopped due to slow patient accrual.^5^ The present data may help to address RR risk in those patients. Given a reported risk of metastatic lymph node involvement and the deployment of these nonsurgical treatment modalities, the subsequent risk of axillary recurrence can be estimated. Another RCT currently investigates whether omitting SLNB in clinically node-negative breast cancer patients undergoing BCT is noninferior to the current axillary staging regimes.^6^ Based on the reported risk of tumor-positive lymph nodes of 12–16% in this ultrasound-negative patient category, the subsequent risk of RR can be estimated in relation to the nonsurgical treatments.

## References

[1] Nieweg OE, Jansen L, Valdes Olmos RA (1999). Lymphatic mapping and sentinel lymph node biopsy in breast cancer. Eur J Nucl Med.

[2] Armando E, Giuliano AE, Ballman KV (2017). Effect of axillary dissection vs no axillary dissection on 10-year overall survival among women with invasive breast cancer and sentinel node metastasis: the ACOSOG Z0011 (Alliance) randomized clinical trial. JAMA..

[3] Roos MM, van Steenhoven JEC, Aalders KC (2019). Regional recurrence risk following a negative sentinel node procedure does not approximate the false-negative rate of the sentinel node procedure in breast cancer patients not receiving radiotherapy or systemic treatment. Ann Surg Oncol..

[4] van Steenhoven JEC, Kuijer A, van Maaren MC, et al. Quantifying the mitigating effects of whole breast radiotherapy and systemic treatments on regional recurrence incidence in breast cancer patients. *Ann Surg Oncol*. 2020. 10.1245/s10434-020-08356-2. 10.1245/s10434-020-08356-2 PMC7410865 32198570

[5] Roozendaal LM, de Wilt JH, van Dalen T (2015). The value of completion axillary treatment in sentinel node positive breast cancer patients undergoing a mastectomy: a Dutch randomized controlled multicentre trial (BOOG 2013-07). BMC Cancer..

[6] Roozendaal LM, Vane MLG, van Dalen T (2017). Clinically node negative breast cancer patients undergoing breast conserving therapy, sentinel lymph node procedure versus follow-up: a Dutch randomized controlled multicentre trial (BOOG 2013-08). BMC Cancer..

